# When do toddlers point during mealtime?: Pointing in the second year of life in everyday situations

**DOI:** 10.3389/fpsyg.2023.1050975

**Published:** 2023-01-26

**Authors:** Jun Kasuya, Tetsushi Nonaka

**Affiliations:** Graduate School of Human Development and Environment, Kobe University, Kobe, Japan

**Keywords:** pointing, shared intentionality, caregiver–child interactions, social cognition, mealtime

## Abstract

The present study aimed to gain insight into the development of the infant’s awareness of others’ attention that takes place in everyday contexts. We examined the relation between the toddler’s pointing, the toddler’s visual attention to the caregiver, and the context of the action of the caregiver in the same child-caregiver dyads at two time points (13 and 17 months of age) during lunchtime at a Japanese daycare center, in which toddlers ate lunch with the help of caregivers. Specifically, we focused on the question of whether the timing of the toddler’s pointing reflected the ongoing context of the action of the caregiver, based on the analysis of what the caregiver was doing when a toddler exhibited pointing behavior. Our analysis revealed several interrelated results. First, the toddler’s pointing behavior was related to the visual exploration of the face of the caregiver at 17 months of age, which was not obvious at 13 months of age. Second, toddlers were more likely to point when the caregivers were just looking at them without being engaged in other salient goal-directed activities. Third, toddlers were less likely to exhibit pointing behavior when the caregivers were manipulating objects or feeding the toddlers. Taken together, the results suggested that toddlers were increasingly aware of the dynamic context of social partner’s engagement, differentiating the right time to modulate the attention of others by pointing in everyday situations. The present study supplemented the existing knowledge about pointing and the development of shared intentionality based on controlled experiments by providing a description of the context in which toddlers tend to point in the naturalistic situation of lunchtime within a specific cultural setting during the second year of life.

## 1. Introduction

Understanding the attention and intention of others in a populated environment is one of the key psychological properties of humans. Around the end of the first year, human infants are known to start sharing experiences with familiar persons by pointing to objects of joint interest, using vocalizations and gestures, and attending to and imitating expressions and behaviors (Carpenter et al., 1998; Trevarthen, 1998, 2001, 2002). Pointing, among others, is a form of communication that induces the other person to attend to some particular external entity (Tomasello et al., 2007). Unlike other symbolic gestures, pointing does not convey a specific meaning by itself. By drawing the other person’s attention to the object to which the actor is pointing, the actor can communicate a number of meanings regarding that object to the other person, and the development of pointing has been considered to be closely related to the development of language (Tomasello and Farrar, 1986; Tomasello, 2003; Goldin-Meadow and Butcher, 2003; Goldin-Meadow, 2007; Goldin-Meadow and Alibali, 2013).

Previous studies have often considered the issue of infant pointing by classifying pointing behavior into two broad categories (Bates et al., 1975): protoimperative pointing and protodeclarative pointing. Protoimperative pointing is a pointing behavior that uses an adult as a tool to obtain an object. Protodeclarative pointing is a pointing behavior that uses the object that is pointed as a tool to gain the attention of an adult. Accordingly, the theoretical debates about infant pointing have often centered on the question of whether infant pointing is controlled in such a way to inform others about something or whether, alternatively, it is simply controlled to influence the behavior of others (e.g., Camaioni, 1993; Moore and D'Entremont, 2001; Camaioni et al., 2004; Cochet and Vauclair, 2010; Sodian and Kristen-Antonow, 2015; Salo et al., 2018).

Tomasello et al. (2007) suggested that the actual situation might be more complex than such a dichotomy. For example, when pointing, an infant wants adults to respond to both the infant and the pointing object, not just either the infant or the objects (Liszkowski et al., 2004), indicating that in protodeclarative pointing, infants may not only direct attention of others to a particular aspect of external entities but can also seek to share an attitude with an adult about a common referent. Likewise, infants appear to understand the intention of others even though their pointing appears goal-directed and purpose-driven (Golinkoff, 1993). Based on experimental evidence, Tomasello et al. (2007, p. 208) argued that communicative pointing requires an understanding of intentions, shared attention and knowledge, and that the emergence of pointing around the end of the first year marks the significant development of understanding of the formula “*she intends that I attend to X*” relevant to some joint attentional common ground.

By contrast, Reddy (2010) viewed the emergence of pointing not as a result of a qualitative shift in understanding the attention of others, but as part of a continuum of expanding awareness of what kind of things in the world others can be engaged with. Infants engage in face-to-face exchanges with other people, are sensitive to direct gaze at themselves, and respond to others’ acts toward them with recognizable responses, noticing when others look at them, and imitating actions themselves to call others from 2 months. Gradually, infants become able to use little tricks and funny movements to attract and retain the attention of other people well before they engage in protodeclarative pointing (Reddy, 2010). Based on the observation that the infant changes from calling attention to the self as a whole to calling attention to particular actions of her body, and then to external things through the first year, Reddy claimed that the awareness of attention develops not through a discovery of covert entity hidden behind the behavior (implied by such terms as “theory of mind,” c.f., Wellman, 2014), but through developing awareness of the scope of others’ overt engagement with some object or fact of the environment in specific situations (see also Markova and Legerstee, 2015; Nomikou et al., 2017).

According to this latter view, a systematic study of the natural ecology of infants pointing is required to understand the process of differentiation, expansion, and refinement of the infant’s awareness of attention in a specific cultural setting. For attending is fundamentally related to its objects, where the awareness of other people’s attending is necessarily related to the awareness of its objects in its context. It has been suggested that normally-occurring daily interactions between parents and infants provide the foothold for infants to tune into social information and learn to coordinate attention with their partners (Bakeman and Adamson, 1984). Csibra (2003) showed that 12-month-old infants’ understanding of the actions of others is sensitive to the specific communicative situations in which these actions occur. Such fine attunement to specific contexts goes hand in hand with the development of the exploratory activity of infants. From around 10 months of age, infants pay attention to the looking behavior of social partners to establish a common ground for smooth interaction (Corkum and Moore, 1995; Brooks and Meltzoff, 2005; Frischen et al., 2007). In the second year of life, children become more likely to look at their communicator before pointing at 18 months of age compared to at 12 months of age (Franco and Butterworth, 1996), suggesting that exploratory activity directed to the objects of mutual attention in the environment continues to be refined after the first instances of pointing. It is also known that the development of mutual awareness of attention between infants and caregivers is specific to cultural contexts. For example, the studies that compared Scottish and Japanese infant-caregiver dyads have shown that cultural difference exists in the style of co-regulation of behavior based on the awareness of each other’s attention in daily activities such as feeding (Negayama et al., 2021) and negotiation of inter-personal distance (Negayama and Trevarthen, 2022).

In the present study, we aimed to gain insight into the process of differentiation, expansion, and refinement of the toddler’s awareness of others’ attention that takes place in a specific cultural context of mealtime, by focusing on the pointing and looking behavior of toddlers during lunchtime at a Japanese daycare center. We analyzed the same child-caregiver dyads at two time points (13 months and 17 months of age) in their daily situations at lunchtime. Specifically, we examined the question of whether the timing of the toddler’s pointing reflected the ongoing context of the action of the caregiver, based on the analysis of what the caregiver was doing when a toddler exhibited pointing behavior. Thereby, we further aimed to describe the context in which toddlers tend to point in the naturalistic situation of lunchtime within a specific cultural setting during the second year of life.

## 2. Materials and methods

### 2.1. Participants

Six toddlers (three females and three males) and two adult female caregivers in a daycare center in Japan participated in this study. In the daycare center, each caregiver took care of three children, and each of the two caregivers took care of the three toddlers that participated in the study. Data were extracted from video recordings of toddlers as they ate lunch with the assistance of a caregiver. These recordings were made longitudinally over a period of 10 months from June 2017 to March 2018. All parents and caregivers gave informed consent prior to being filmed. The toddler–caregiver dyads were fixed throughout the observation period, where the same caregiver always looked after the same child. Each of the two caregivers who participated in the study took care of the three children one after another during lunchtime at the daycare. For older children, one caregiver sometimes assisted two children at a time, that is, one child occasionally sat next to another child during a meal, but the interaction between the children was rarely observed.

### 2.2. Procedure

The data were part of a longitudinal project that made monthly multi-day visits to the same children at the daycare, and the analysis of the part of the data from the same video observation material has been previously reported in Nonaka and Stoffregen (2020). A researcher visited the daycare monthly, placing digital video cameras in front of each table so as to record the toddler eating a meal with the caregiver. To avoid distraction, the researcher left the room before the toddlers came in. The toddler sat in his or her usual chair facing a table, and the caregiver sat while holding him/her or around the corner of the same table next to the toddler. To capture the development of communication in toddlers after they started pointing around their first birthdays, we collected the data from the following two periods; when the toddlers were 13 months of age and 17 months of age. Each month, lunchtimes for four consecutive days were videotaped, but not all the children were present for all the observed lunchtime. In this study, we selected the lunchtime of 3 days within the same week where each child was present, in which the age of the child was closest to 13 months or 17 months of age. The mean ages of the toddlers at the two observation periods were 12.96 months (*SD*: 0.60 months) and 17.28 months (*SD*: 0.48 months), for 13 months and 17 months, respectively.

### 2.3. Data coding

Caregiver–toddler interactions were coded from video clips recorded with one frontally oriented camera. All coding from the video clips was completed in frame-by-frame mode using the video coding software *Datavyu*^1^ which allows for frame-by-frame analysis of the timing of onsets and offsets of specific behaviors (i.e., 30 frames per second were visible and available for coding). We observed and analyzed a total of 36 mealtimes of 6 toddlers × 2 periods × 3 times.

**Figure 1:**
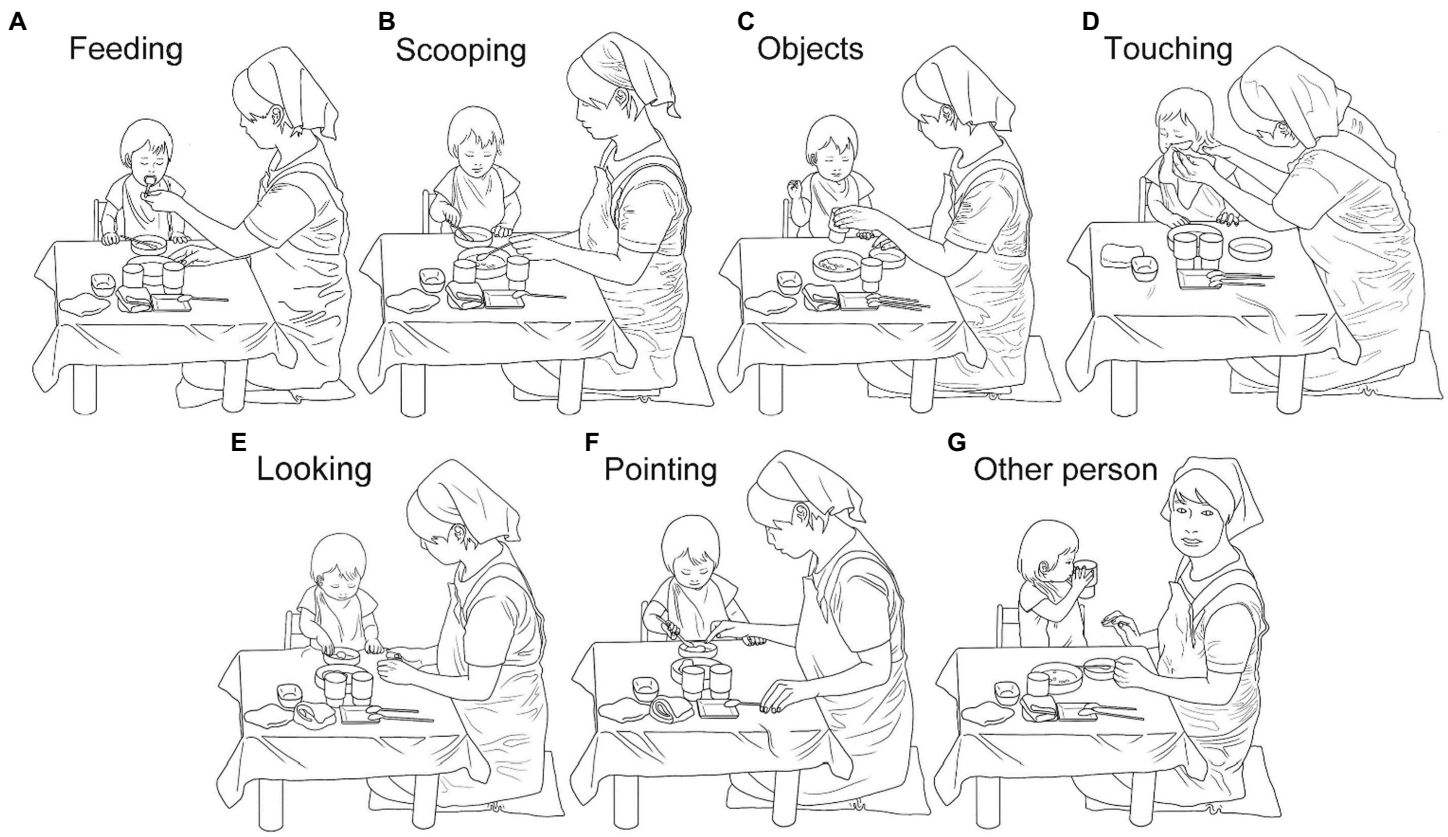
Seven categories of caregiver behaviors used in the analysis; **(A)** feeding: A caregiver feeds a toddler. **(B)** scooping: A caregiver scoops food. **(C)** objects: A caregiver handles objects (e.g., plates or towels). **(D)** touching: A caregiver touches a toddler to assist her/him. **(E)** looking: A caregiver looks at a toddler without being engaged in other observable tasks. **(F)** pointing: A caregiver points at objects on the table. **(G)** other person: A caregiver attends to other toddlers or caregivers.

The observed data was the whole lunchtime, marked by the following two events: The start point was the moment when the infant sat down at the table, and the endpoint was the moment when the infant left the table after finishing lunch. The mean duration of the observed data was 19 min 6 s (*SD*: 3 min 51 s), and 17 min 2 s (*SD*: 3 min 53 s), for 13 months and 17 months, respectively. A primary coder recorded (a) the toddler’s gaze directed at the caregiver’s face, (b) the toddler’s pointing behavior, and (c) the caregiver’s behaviors according to the classification described in what follows. The caregiver’s behaviors were initially classified into ten categories based primarily on their manual interaction, based on the previous report that manipulation of objects is important in establishing joint attention between parents and infants (Yu and Smith, 2013): (1) feeding: A caregiver feeds a toddler. (2) scooping: A caregiver scoops food. (3) objects: A caregiver handles objects (e.g., plates or towels). (4) touching: A caregiver touches a toddler to assist her/him. (5) looking: A caregiver looks at a toddler without being engaged in other observable tasks. (6) pointing: A caregiver points at objects on the table. (7) other person: A caregiver attends to other toddlers or caregivers. (8) gestures: A caregiver makes gestures other than pointing. (9) self: A caregiver acts directed to herself. (10) no data: A caregiver is not seen in the video. As we will report in the Result, among the above ten categories, gestures, self, and no data occurred very rarely in the 36 videos, and to ensure reliability, we focused our analysis on the seven categories of caregiver’s behavior in the subsequent analyzes (Figure 1), excluding gestures, self, and no data.

### 2.4. Data analysis

**Figure 2:**
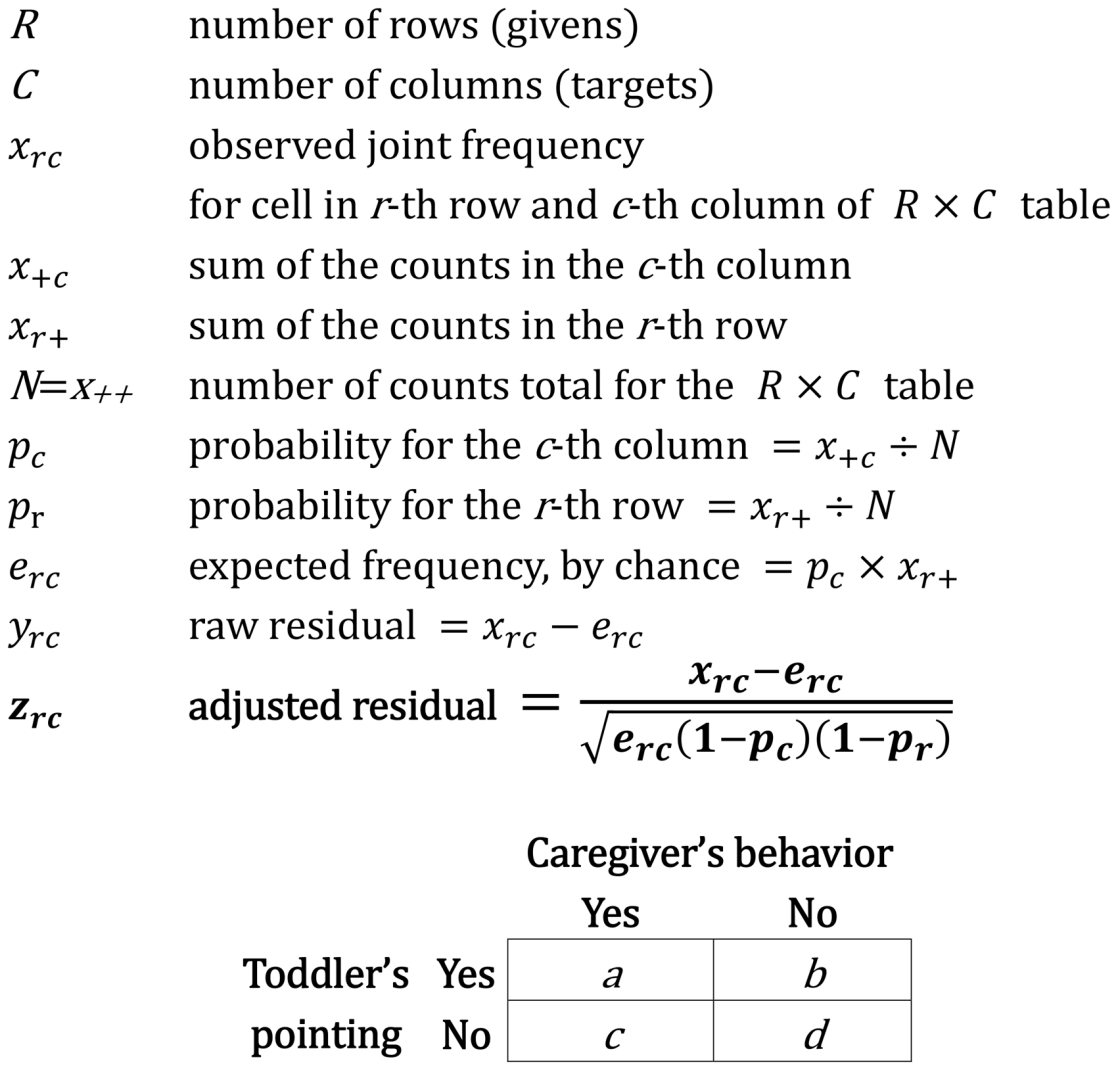
Definition for *z*-score (adjusted residual, *z_rc_*) based on the contingency table used in the present study (Bakeman and Quera, 2011).

We examined the developmental changes in the frequency of the toddler’s gaze directed at the caregiver’s face, the frequency of pointing, and the proportion of simultaneous occurrence of pointing and the looks to the caregiver’s face—defined as looking within the two-second window before and after the pointing following the method used in Franco and Butterworth’s (1996) experiment—at two time points (13 and 17 months of age) in the same dyads. Then we further tested how much more or less likely compared to frequencies if the toddler’s pointing in each category of caregiver’s behavior defined above. For the analysis, we created two-dimensional contingency tables: The rows of the contingency tables were the presence or absence of each category of caregiver’s behavior, while the columns were the presence or absence of toddler’s pointing. In these tables, each cell contained the frequency of the toddler’s pointing and not pointing when the caregiver exhibited a certain behavior or not. Based on the contingency table, we computed *z*-scores based on the difference between observed and expected joint frequencies (Figure 2). The expected frequency for a cell in a contingency table is the probability of its toddler’s point (column; *pc* in Figure 2, the overall rate of the toddler’s pointing behavior) multiplied by the frequency of its caregiver’s behavior (row). The *z*-score reflects the degree to which the observed frequency of each sequence differs from the expected frequency by chance. In other words, the *z*-score reflects the degree to which a caregiver’s behavior occurred (or not) as a condition of a toddler’s pointing, taking into consideration the base rate of the actions in the sequence (*pc* and *pr* in Figure 2). In doing so, the *z*-score “controls” for the total number of occurrences for each behavior and allows for the probability values to be reasonably assessed. The *z*-score is positive if the observed is greater than chance and negative if the observed is less than chance. If there is no association between the caregiver’s behaviors and the toddler’s points, then *z*-scores would be distributed approximately normally with a mean of 0 and variance equals to 1. Overall, a large *z*-score indicates a greater-than-expected occurrence (relative to chance) of that sequence (Bakeman and Quera, 2011).

To compare the occurrence of sequences with chance, the probabilities of each meal (i.e., individual *z*-scores obtained for the 36 mealtimes from 6 participants) were used as dependent variables and tested whether *z*-scores were statistically different from zero by the one-sample *t*-test. If the occurrence of pointing action was not related to the caregiver’s behavior, the mean *z*-score would hover around 0 and therefore not differ from 0. In addition, using the *lme* function in the *nlme* package of the R statistical software (Pinheiro and Bates, 2019), *z*-scores, the frequency of pointing, and the frequency of looking at the face of the caregiver were modeled using a linear mixed-effects model. The fixed effects factors were periods (13 months and 17 months) and toddler was included as a random effect for the intercept as well as its slope with respect to period. To model heteroscedasticity, we used a variance function (*varIdent* of *nlme* package) that allowed different variances per stratum for individual participants. In addition, the correlation between the frequency of pointing and looking at the face of the caregiver observed at each mealtime was computed separately for 13 and 17 months. For all statistical tests, we used criterion *α* = 0.05 (two-sided). In addition to the pooled data from the six children, the data of each individual child are also presented in the supplementary material (Supplementary Table S1).

## 3. Results

### 3.1. Descriptive statistics for toddler and caregiver actions

Descriptive statistics for the toddler and caregiver actions are presented in Table 1. On average, the mealtime of children lasted for 19 min 6 s (*SD* = 3 min 51 s) at 13 months and 17 min 2 s (*SD* = 3 min 53 s) at 17 months. During mealtime, the toddlers performed, on average, frequency of pointing and the gaze directed at the face of the caregiver were 13.28 (*SD* = 17.34) and 15.33 (*SD* = 9.77) at 13 months, respectively. At 17 months, both the frequency of pointing and that of gaze directed at the caregiver’s face increased to 18.89 (*SD* = 16.26) and 23.94 (*SD* = 19.14), respectively. The frequency of ten categories of the behavior of the caregivers observed during mealtime is shown in Table 1. In both time points, during toddlers’ mealtime, caregivers were mostly engaged in meal-related actions such as feeding, scooping, and moving objects (such as plates), or just watching over or assisting the toddler. Gestures and other actions that are not directly related to feeding the toddler were observed only a few times (Table 1).

**Table 1:** Mean and standard deviation of meal time, age of the toddler, the frequencies of toddler’s pointing and face looking, and the ten categories of caregiver’s behavior observed within each meal when the toddler was 13 and 17 months old.

	13 months	17 months
	*M* (SD)	*M* (SD)
Mealtime duration	19 m 6 s (3 m 51 s)	17 m 2 s (3 m 53 s)
Toddlers (*n* = 6)		
Age (month)	12.96 (0.60)	17.28 (0.48)
Pointing (frequency)	13.28 (17.34)	18.89 (16.26)
Face looking at a caregiver (frequency)	15.33 (9.77)	23.94 (19.14)
Caregivers’ (*n* = 2) behavior (frequency)		
Feeding	40.61 (10.59)	27.06 (11.47)
Scooping	55.50 (14.23)	49.06 (10.31)
Objects	53.78 (11.17)	42.22 (13.32)
Touching	29.61 (10.77)	24.00 (10.24)
Looking	59.11 (22.85)	45.78 (14.98)
Pointing	5.61 (7.51)	7.78 (9.01)
Other person	9.94 (5.02)	13.44 (8.93)
Gestures	3.78 (2.22)	3.11 (2.31)
Self	2.50 (1.71)	3.00 (2.56)
No data	0.06 (0.23)	0.94 (1.47)

Three categories (*gestures*, *self*, and *no data*, below the dotted line) that were not frequently observed were excluded from the analysis.

### 3.2. Changes in the frequency of pointing and looking at the caregiver’s face

**Figure 3:**
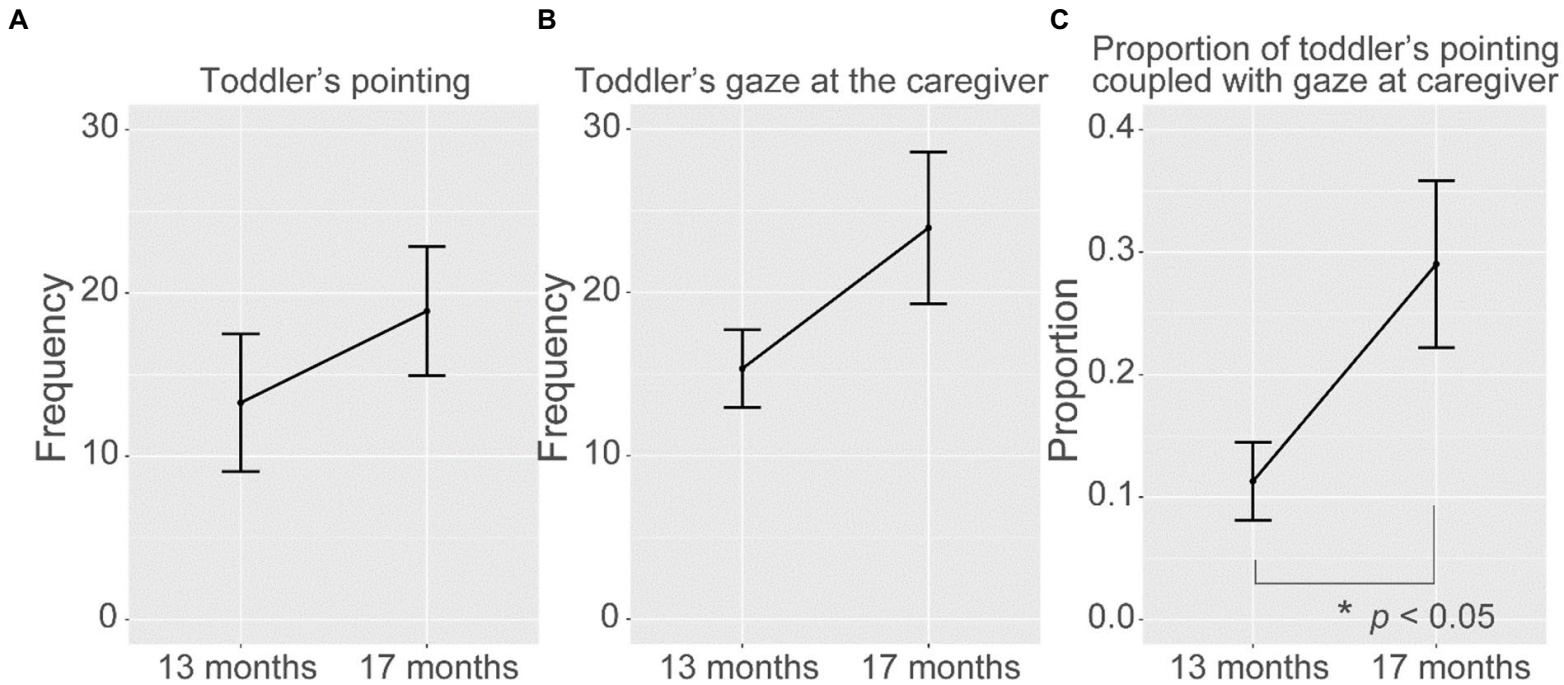
Differences in the frequency of **(A)** toddler’s pointing and **(B)** toddler’s gaze directed at the face of the caregiver between 13 and 17 months of age. **(C)** The proportion of pointing in which the infant looked at the caregiver during the time window of 2 s before and after the toddler pointed. The figure is based on the data from the three observations of lunchtime for six children at two time points (13 months and 17 months of age). Error bars represent the standard error of the mean.

To gain insight into the attention of toddlers, we examined the frequency of pointing by the toddlers and how it changed over time. Visual inspection of Figure 3A suggested that the frequency of pointing tended to increase, but there was no statistically significant effect of period (13 months vs. 17 months) on the frequency of pointing (*F*_(1, 29)_ = 0.86, *p* = 0.36). We also examined the frequency of toddlers’ looks at the caregiver’s face, which exhibited a similar tendency but was not statistically significant as well (*F*_(1, 29)_ = 2.30, *p* = 0.14; Figure 3B). When we further looked into the relation between the frequency of pointing and that of looking at the caregiver’s face at each meal time observed, when toddlers were 13 months old, there was no correlation between the frequency of toddler’s pointing and that of toddler’s looks at the caregiver (*r*_16_ = 0.13, *p* = 0.59). However, when they turned 17 months old, the frequency of toddlers’ pointing exhibited a significant correlation with the frequency of looks at the caregiver (*r*_16_ = 0.66, *p* = 0.003). Therefore, from 13 to 17 months of age, the frequency of pointing became significantly related to the frequency of looking at the caregiver’s face for each mealtime, implying that toddlers pointing behavior were coupled with the visual exploration of the states of the caregiver in 17 months of age, which was not obvious in 13 months of age.

In order to more clearly ascertain the connection between the pointing and the gaze on the caregiver’s face, we further calculated and compared the proportion of pointing in which the infant looked at the caregiver during the time window of 2 s before and after the infant pointed, at each time point of 13 and 17 months. The results are shown in Figure 3C. The analysis found a statistically significant increase in the proportion of pointing with attention to the caregiver’s face before and after pointing from 13 to 17 months (*F*_(1, 27)_ = 5.12, *p* < 0.05). The result indicated that not only the frequency of pointing and gaze directed at the face of the caregiver, but also the temporal contingency between these two actions got stronger across the two time points.

### 3.3. Caregiver’s behaviors at the time when toddlers pointed

**Figure 4:**
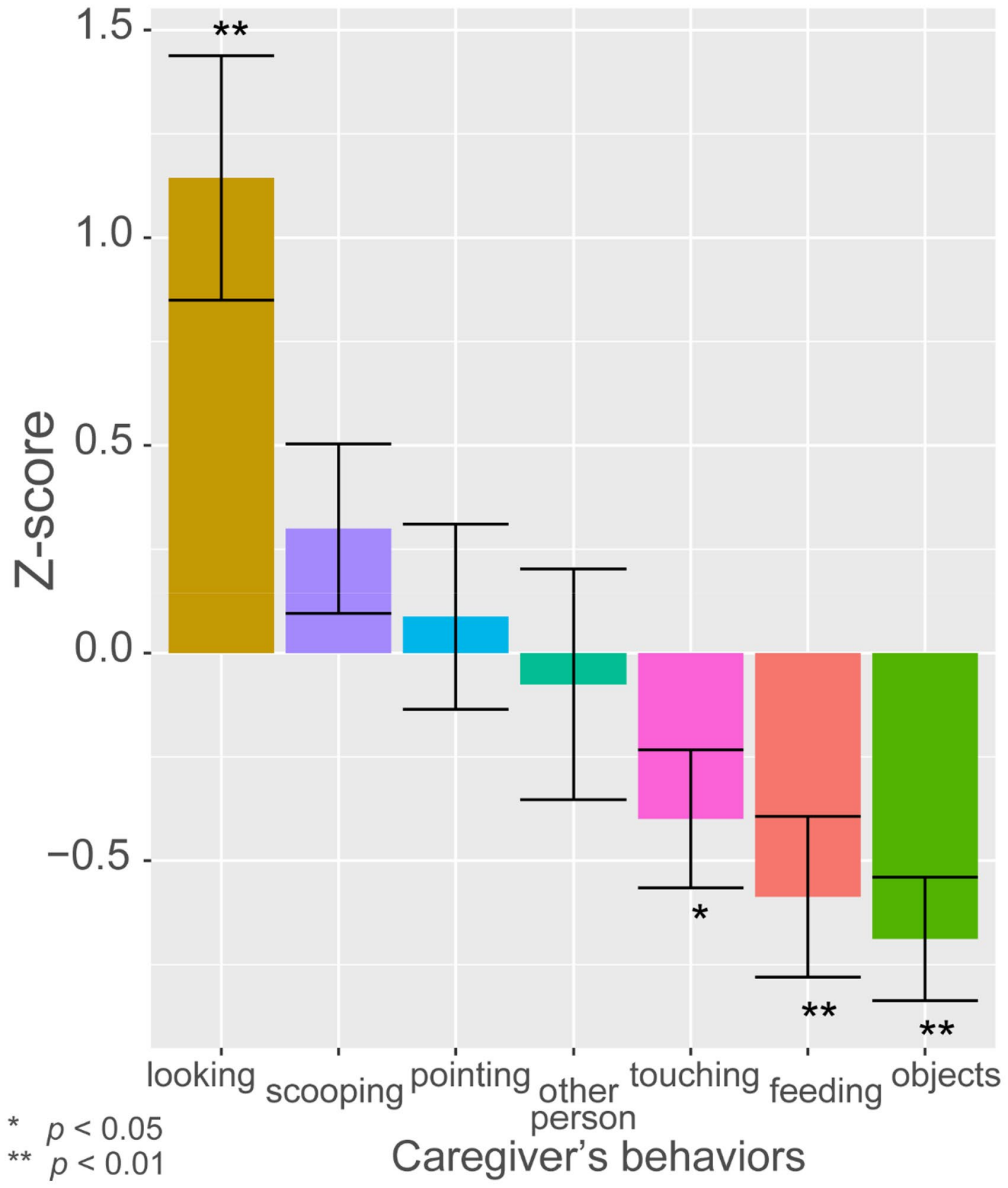
The *z*-scores of the difference between the expected frequency and the observed frequency of toddlers’ pointing during each category of caregiver’s behaviors. The figure is based on the data from the three observations of lunchtime for six children at two time points (13 months and 17 months of age). Error bars represent the standard error of the mean.

To investigate whether the toddlers’ points were influenced by the caregivers’ behaviors, we examined what the caregiver was doing at the moment when a toddler exhibited pointing behavior. We computed the *z*-scores of the difference between the expected frequency and the observed frequency of toddlers’ pointing behavior during each of the 10 classifications of caregiver’s behavior (Figure 4). One-sample *t*-test found that toddlers were significantly more likely than chance to point when caregivers were just looking at them, *t*(33) = 3.9, *p* < 0.001. On the contrary, toddlers were significantly less likely to point when caregivers were touching the toddler to help (*t*(33) = −2.4, *p* < 0.05), feeding the toddler (*t*(33) = −3.0, *p* < 0.01), or manipulating objects (*t*(33) = −4.6, *p* < 0.0001; Figure 4). There was no significant difference between the likelihood of pointing and chance when the caregiver was scooping food (*t*(33) = 1.5, *p* = 0.15) and pointing (*t*(30) = 0.39, *p* = 0.70). The result demonstrated that the timing of toddler’s pointing behavior was clearly influenced by the context of action the caregiver was in, where toddlers exhibited pointing behavior more frequently when the caregiver was paying attention to them, corroborating the idea that children in this period understand what their social partners were engaged in and attending to in the complex, natural context of mealtime.

### 3.4. Developmental changes the timing of toddler’s pointing

**Figure 5:**
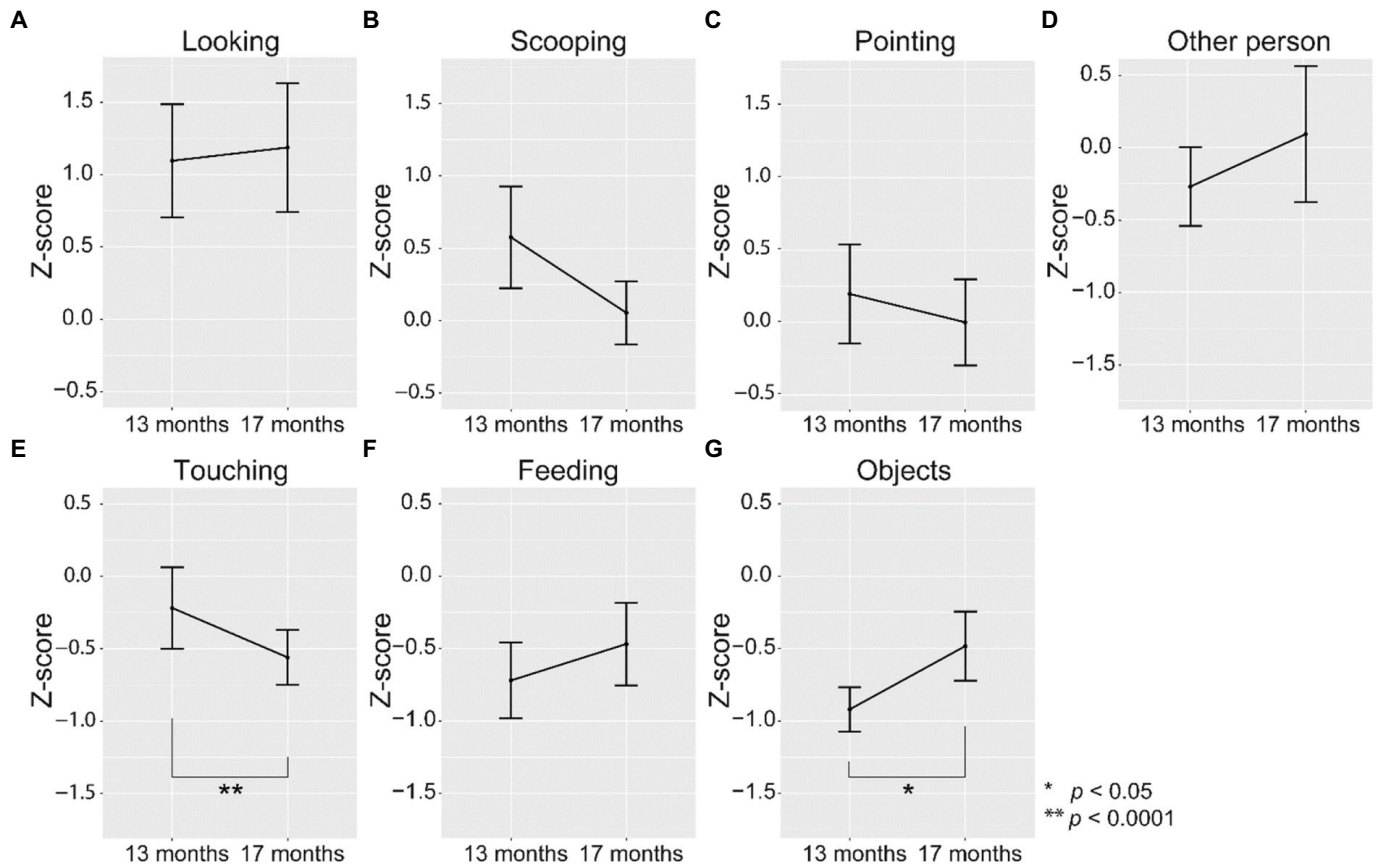
Comparison of *z*-scores between 13 and 17 months **(A)** when the caregiver did nothing and just looked at the toddler, **(B)** when the caregiver was scooping food, **(C)** when the caregiver was pointing, **(D)** when the caregiver acts for other toddlers or caregivers, **(E)** when the caregiver was touching the toddler with contact, **(F)** when the caregiver was feeding the toddler, and **(G)** when the caregiver was manipulating the object. The figure is based on the data from the three observations of lunchtime for six children at two time points (13 months and 17 months of age). Error bars represent the standard error of the mean.

As we mentioned previously, toddlers pointing behavior became coupled with the visual exploration of the face of the caregiver in the 17th month, but not so in the 13th month. To consider whether such a change is reflected in the timing of the toddler’s pointing, we further looked into developmental changes in the relation between the toddler’s pointing and the caregiver’s behavior (Figure 5). A mixed-effects model ANOVA found a highly significant effect of the period (13 vs. 17 months) on the likelihood of pointing during touching by caregivers (*F*_(1, 27)_ = 62.69, *p* < 0.0001), which showed that toddlers were less likely to point while the caregiver were touching toddlers to assist them when they were 17 months old, compared to when they were 13 months old. There was also a marginal effect of period on the likelihood of pointing when caregivers were manipulating objects (*F*_(1, 27)_ = 5.54, *p* < 0.05), although in both periods, toddlers were less likely to point when caregivers were manipulating objects. No significant differences were found in other behaviors. There was a developmental change in certain aspects of toddlers’ pointing behavior in relation to the context of the action of caregivers between 13 and 17 months of age, which implied a subtle change in the toddlers’ awareness of the objects of the caregiver’s engagement in a specific context of mealtime.

## 4. Discussion

In the present study, we examined video recordings of toddlers eating with the help of a caregiver. In order to understand the expansion and differentiation of the infant’s awareness of the objects of others’ attention, we analyzed pointing behavior observed during everyday contexts of mealtime in the second year of life—at 13 and 17 months of age—at a Japanese daycare center. Specifically, we examined the question of whether the timing of the toddler’s pointing reflected the ongoing context of the action of the caregiver, based on the analysis of what the caregiver was doing when a toddler exhibited pointing behavior. We also asked how the coupling between toddlers’ pointing and their gaze directed at the caregiver during a meal changed over time. Our analysis revealed several interrelated results. First, the toddler’s pointing behavior was related to the visual exploration of the face of the caregiver at 17 months of age, which was not obvious at 13 months of age. Second, toddlers were more likely to point when the caregivers were just looking at them without being engaged in other salient goal-directed activities. Third, toddlers were less likely to exhibit pointing behavior when the caregivers were manipulating objects or feeding the toddlers. Fourth, the timing of pointing by toddlers varied developmentally, in which the likelihood of pointing when caregivers were touching the toddlers decreased. In what follows, we discuss these results in terms of how toddlers in the second year of life differentiate the objects of caregivers’ attention and communicate with them by pointing in mealtime situations.

### 4.1. Toddler’s pointing and visual attention to the caregiver

The frequency of pointing tended to increase with age, but there was a large variability across observed mealtimes (Table 1) and there was no statistically significant overall increase in the frequency of pointing. Likewise, the frequency of visual attention to the caregiver’s face tended to increase but was so variable that there was no statistical difference across the two time points observed. However, when we looked at the relation between the frequency of pointing and that of the face looking in each meal, a systematic correlation between the frequency of pointing and that of looking at the caregiver’s face emerged only when children were 17 months of age, but not when they were 13 months of age. For pointing to function as a means of communication, shared mutual attention needs to be established (Trevarthen, 2002; Tomasello et al., 2007). It has been pointed out that from about 12 months of age, toddlers flexibly adjust their behavior in such a way to take into account the attention and intention of others when communicating with them (Franco and Butterworth, 1996; Csibra, 2003; Brooks and Meltzoff, 2005; Liebal et al., 2010; Liszkowski, 2014). The emergence of a correlation between the frequency of pointing and that of visual attention to the caregiver and the increase of temporal coupling between the two actions at 17 months of age may indicate that toddlers were becoming increasingly sensitive to the object of others’ attention during the second year of life in the daily situations of mealtime. Toddlers’ pointing, especially at 17 months of age, did not appear to be a one-way request, but rather seemed to be the unfolding of a system in which the action of the toddler was reciprocally coupled to that of the caregiver, whose link was established by the mutual awareness of the shared context of engagement (see Nonaka and Goldfield, 2018 for a related discussion).

### 4.2. Toddlers’ awareness of the objects of caregivers’ engagement

In the present study, toddlers were more likely to point when caregivers were looking at them without being engaged in other salient goal-directed activities. This result suggests that 13-month-old toddlers controlled their communicative behavior according to the transient state of attention of others. The result provides support for the interpretation that toddlers were not simply conveying their needs, but were aware of the objects of the caregiver’s engagement in the dynamic context of mealtime. Ramenzoni and Liszkowski (2016) reported an increase in the frequency of reaching by 8-month-old infants toward inaccessible objects only when others were nearby. Their result showed that infants understood that others were being helpful, and that the object can be the potential object of mutual engagement with social partners. Likewise, the result of the present study indicated that toddlers are discriminating subtle differences in the possibility of mutual engagement. Even though caregivers were engaged in observable goal-directed activities, when caregivers were scooping the food and pointing, the toddlers did not suppress their pointing behavior (Figure 4), presumably because caregivers were responsive to toddlers when they were selecting food to bring to the toddlers. In this respect, the present results may be seen as concrete examples of cooperative imperative in the naturalistic situation of mealtime (Golinkoff, 1993; Tomasello et al., 2007), in which toddlers pointed at the next food they wanted to eat, in a flexible manner based on the understanding of the context of the action of others.

Our analysis further found that the likelihood of pointing by toddlers when caregivers were touching to assist the toddlers decreased from 13 to 17 months. In these situations, the caregivers’ attention was diverted from triadic interactions between food objects and the toddler, but instead was focused on the toddler herself. At 17 months of age, toddlers were likely to be aware of such a shift of caregivers’ attention toward dyadic interaction, and as a result, they might have chosen not to point at things. When caregivers were engaged in activities not related to toddlers or feeding (i.e., “other person” category), the likelihood of the occurrence of toddlers’ pointing was close to chance, probably because caregivers’ attention shifted away from the interaction with toddlers. In these situations, toddlers sometimes pointed to get their attention back, while at other times they chose not to point. The situation may be similar to that in Liszkowski et al.’s experiment (2004), where adult responses to infant pointing were manipulated. Infants appeared satisfied only when adults responded to both the pointed object and them, and they showed dissatisfaction when adults responded only to pointed objects or only to them, or when adults did not respond, by repeatedly pointing or stopping pointing. Likewise, the results of the present study seem to provide support for the idea that what matters in communication by means of pointing is the triadic interaction between the child, the caregiver, and the object of mutual engagement, but not the individual constituent *per se*.

## 5. Conclusion

In the present study, we examined the relation between toddlers’ pointing, toddlers’ visual attention to the caregiver, and the context of the action of the caregiver during lunchtime at a Japanese daycare center at 13 and 17 months of age. In feeding situations, toddlers selectively pointed when the caregiver was looking at the toddlers without being engaged in other obvious tasks, which in turn suggested that toddlers were aware of the dynamic context of other people’s engagement, differentiating the right time to modulate the attention of others by pointing. We also found that pointing by toddlers became increasingly coupled with the visual exploration of the situation of social partners between 13 and 17 months of age. Taken together, the present study demonstrated that children before the age of two, who are not yet proficient in verbal communication, exhibit differentiation and expansion of awareness of the dynamic context of the action of others in everyday situations. The present study supplemented the existing knowledge about pointing and the development of shared intentionality based on controlled experiments by providing a description of the context in which toddlers tend to point in the naturalistic situation of lunchtime within a specific cultural setting during the second year of life.

## Data availability statement

The raw data supporting the conclusions of this article are available on request from the corresponding author. The data are not publicly available due to privacy restrictions.

## Ethics statement

The studies involving human participants were reviewed and approved by Kobe University. Written informed consent to participate in this study was provided by the participants’ legal guardian/next of kin.

## Author contributions

JK and TN designed the study, wrote and reviewed the manuscript. TN collected the data. JK analyzed the data. All authors contributed to the article and approved the submitted version.

## Funding

This work was supported by grants from JSPS KAKENHI Grant Numbers JP21KK0182 and JP22H00988 from the Japan Society for the Promotion of Science, awarded to TN.

## Conflict of interest

The authors declare that the research was conducted in the absence of any commercial or financial relationships that could be construed as a potential conflict of interest.

## Publisher’s note

All claims expressed in this article are solely those of the authors and do not necessarily represent those of their affiliated organizations, or those of the publisher, the editors and the reviewers. Any product that may be evaluated in this article, or claim that may be made by its manufacturer, is not guaranteed or endorsed by the publisher.

## Footnotes

^1^ www.datavyu.org
