# Supplementary material for: When do toddlers point during mealtime?: Pointing in the second year of life in everyday situations
Source: Front Psychol. 2023 Jan 26;14:1050975. doi: 10.3389/fpsyg.2023.1050975 (PMC9909215; doi:10.3389/fpsyg.2023.1050975)
Supplement: Supplementary file 1 [file Table_1.pdf]

Table S1. The individual data of the frequency of toddler's pointing, toddler's gaze directed at the face of the caregiver, the proportion of pointing in which the infant looked at the caregiver during the time window of 2 seconds before and after the infant pointed, and the *z*-scores of the difference between the expected frequency and the observed frequency of toddlers' pointing during each category of caregiver's behaviors between 13 and 17 months of age.

	Toddler 1		Toddler 2		Toddler 3		Toddler 4		Toddler 5		Toddler 6	
Month	13	17	13	17	13	17	13	17	13	17	13	17
	M (*SD*)	*M* (*SD*)	*M* (*SD*)	*M* (*SD*)	*M* (*SD*)	*M* (*SD*)	*M* (*SD*)	*M* (*SD*)	*M* (*SD*)	*M* (*SD*)	*M* (*SD*)	*M* (*SD*)
Frequency of pointing	1.67 (1.70)	44.33 (11.09)	13.67 (2.62)	21.67 (9.57)	10.67 (8.58)	7.33 (3.30)	12.00 (6.48)	5.67 (3.30)	40.00 (26.20)	30.67 (3.09)	1.67 (0.94)	3.67 (2.49)
Frequency of face looking at a caregiver	4.33 (2.87)	49.67 (10.66)	28.67 (6.13)	42.00 (18.71)	20.00 (3.27)	6.67 (3.30)	19.67 (3.30)	24.00 (5.35)	15.33 (6.13)	13.00 (5.66)	4.00 (0.00)	8.33 (3.86)
Proportion of pointing with face looking	0.00 (0.00)	0.48 (0.14)	0.25 (0.09)	0.32 (0.16)	0.28 (0.10)	0.12 (0.09)	0.09 (0.07)	0.30 (0.36)	0.08 (0.02)	0.14 (0.06)	0.00 (0.00)	0.38 (0.44)
Z-scores of the difference between the expected and observed frequency of toddlers' pointing for each caregiver's behavior												
Looking	0.34 (1.32)	3.14 (2.52)	1.73 (0.77)	2.34 (1.68)	3.34 (1.33)	-0.35 (0.95)	0.76 (1.33)	0.85 (0.48)	1.40 (0.70)	0.62 (0.67)	-0.51 (0.11)	0.53 (1.03)
Scooping	0.31 (0.75)	0.31 (0.26)	-0.05 (0.22)	0.03 (0.94)	0.39 (1.57)	-0.98 (0.45)	2.38 (0.86)	1.03 (0.47)	0.18 (1.18)	0.19 (1.00)	0.09 (1.25)	-0.27 (0.55)
Pointing	-0.12 (0.01)	-1.04 (0.57)	-0.26 (0.32)	-0.19 (0.56)	-0.62 (0.33)	-0.38 (0.21)	-0.49 (0.22)	-0.16 (0.06)	2.24 (1.60)	0.74 (1.27)	-0.09 (0.01)	2.99 (0.00)
Other person	-0.17 (0.04)	-1.47 (0.43)	-0.80 (0.21)	-0.69 (0.10)	-0.16 (0.59)	2.69 (2.28)	-0.58 (0.21)	-0.67 (0.14)	0.50 (2.14)	-0.90 (0.57)	-0.32 (0.07)	1.61 (1.76)
Touching	-0.46 (0.09)	-1.39 (0.11)	0.95 (0.32)	-0.50 (0.58)	-0.01 (0.85)	-0.04 (0.45)	-1.05 (0.21)	-0.27 (0.71)	-1.25 (0.20)	-0.65 (1.24)	0.51 (1.38)	-0.50 (0.17)
Feeding	0.39 (0.78)	-1.35 (0.85)	-0.85 (0.56)	-0.22 (1.36)	-1.63 (0.40)	-0.71 (0.08)	-1.19 (0.19)	-1.04 (0.38)	-1.29 (0.51)	1.22 (1.19)	0.33 (1.14)	-0.71 (0.26)
Objects	-0.79 (0.18)	0.25 (1.37)	-1.12 (0.67)	-1.16 (0.29)	-1.63 (0.12)	-0.15 (0.50)	-1.07 (0.50)	-0.17 (0.32)	-0.90 (0.07)	-1.12 (1.35)	-0.20 (0.46)	-0.54 (0.30)
